# Optimizing reference gene selection for accurate gene expression analysis in plants

**DOI:** 10.1080/15592324.2025.2597054

**Published:** 2025-12-09

**Authors:** Zili Xiong, Cunnian Fu, Shaoyong Huang, Jianlei Shi

**Affiliations:** a Southern Zhejiang Key Laboratory of Crop Breeding, Wenzhou Vocational College of Science and Technology, Wenzhou, People's Republic of China

**Keywords:** Reference genes, expression analysis, multi-omics, transcriptomics, qRT-PCR

## Abstract

Accurate selection of reference genes is crucial for reliable gene expression analysis in plants. Traditional reference genes, such as *GAPDH* and *ACT*, are widely used but often show variable stability under different conditions, stresses, tissue types, and developmental stages. Recent advances in multi-omics technologies, including transcriptomics, offer new approaches for improving reference gene selection. These methods allow for the integration of diverse datasets to identify genes with stable expression across various environmental stresses and developmental stages, providing more robust and context-specific normalization controls. High-throughput sequencing technologies, such as RNA-seq, have enabled the large-scale identification of stable reference genes, and further enhanced this process by correlating gene expression at the transcript level. Additionally, the application of computational tools helps researchers optimize reference gene selection, making the process more efficient and standardized. Personalized and condition-specific reference gene databases are emerging as valuable resources for selecting the most appropriate genes based on experimental conditions. This paper explores the current trends and challenges in reference to reference gene selection and the potential of a transcriptomics approach to address these challenges. The use of these advanced methods will increase the accuracy and reliability of plant gene expression studies, accelerate discoveries in crop improvement and stress resilience in plant biology and agricultural sciences.

## 1 Introduction

Accurate gene expression analysis is fundamental for understanding plant development, metabolic, and stress responses in plant molecular biology research.^1^ Real-time quantitative PCR (qRT-PCR) is one of the most reliable and widely applied techniques, known for its high sensitivity and quantitative precision.^2^ However, the accuracy of qRT-PCR results were influenced by RNA integrity, reverse transcription efficiency, and environmental variations.^3^ Therefore, normalization using validated reference genes is essential for reproducible and meaningful data interpretation.^4^

Reference genes are endogenous genes that exhibit stable expression across different tissues, developmental stages, and environmental conditions.^5^ These genes are involved in basic cellular metabolism and structural maintenance processes and, in theory, are not affected by experimental treatments or environmental factors. Classic reference genes such as *GAPDH*, *ACT*, and *18S rRNA* have been widely adopted for normalization; however, increasing evidence indicates that their stability is context-dependent.^5^ With the rapid expansion of transcriptomic and multi-omics datasets, large-scale screening of condition-specific reference genes has become feasible, improving both accuracy and reproducibility.

Under different stresses or tissues, expression of these classic reference genes fluctuates significantly.^6–9^ Particularly under environmental stress conditions (such as drought, salinity, low temperature, and pests), the expression stability of different reference genes shows notable differences, which is particularly significant in studies of stress/development responsive gene expression.^10-15^ For example, under salt stress, the expression stability of *ACT* and *18S rRNA* is poor in certain plants, while some novel candidate reference genes, such as *eIF4α* and *EF1α*, show higher stability across multiple stress conditions.^10-16^ Therefore, systematically screening for suitable reference genes under specific conditions is crucial for ensuring the reliability of results.

At present, various software tools and methods (such as GeNorm, NormFinder, and BestKeeper) have been developed for evaluating the stability of reference genes.^2^^,^^17^^,^^18^ These tools allow researchers to identify reference genes with stable expression under specific conditions based on experimental data, thereby reducing errors caused by unstable reference gene expression.^2^^,^^17^^,^^18^ Additionally, with the development of high throughput sequencing technologies, an increasing amount of omics data is being used to identify new candidate reference genes.^5^^,^^14^^,^^19^ For instance, using transcriptomic data to screen reference genes has made significant progress in plants such as rice and wheat, providing valuable resources for the selection of reference genes in different species.^7^^,^^19^

The present review reviews the recent research progress in the selection of plant reference genes, focusing on the expression stability of reference genes in different plant species, tissue types, developmental stages, and stress conditions. By analyzing the selection strategies and evaluation methods for different reference genes, we provide a reference for the screening of reference genes in plant molecular biology research and discuss the potential applications of reference gene selection in multi-omics data and molecular marker-assisted screening in the future.^20^^,^^21^

## 2 Definition and role of plant reference genes

Reference genes are usually involved in essential biological processes such as glycolysis, cytoskeleton formation, and ribosome synthesis.^5^ In gene expression studies, reference genes are commonly used as normalization controls to correct for RNA extraction efficiency, reverse transcription efficiency, and errors in qRT-PCR, thereby improving the reliability and accuracy of the data.^22-25^ An ideal reference gene should maintain a stable expression level under different conditions and should not change even under environmental stress or treatment.^26-28^ However, recent evidence suggests that even traditional housekeeping genes such as *ACT* and *GAPDH* exhibit instability under specific abiotic or biotic stress conditions.^15^^,^^16^ Therefore, the concept of a “universal reference gene” has been replaced by the need for context-specific validation. To minimize bias, modern studies often combine multiple reference genes and evaluate their stability statistically using algorithms such as GeNorm, NormFinder, and BestKeeper.^2^^,^^17^^,^^18^

## 3 Commonly used plant reference genes and their functions

Commonly used reference genes include structural protein-coding genes such as *ACT* and α-tubulin (*TUA*), glycolysis-related genes such as *GAPDH*, translation-related genes such as *EF1α*, and ribosomal RNA genes (e.g., *18S* and *26S rRNA*).^10^^,^^29^^,^^30^ These genes play key roles in cellular metabolism and are considered to have stable expression under various conditions (Table 1). *ACT* and *TUA* are structural proteins widely used in plant gene expression research. *ACT* plays a key role in cytoskeleton formation and cell movement, while *TUA* is a component of microtubules and is essential for cell division and elongation. Owing to their important roles in maintaining and dynamically changing cell structure, these genes are used as stable references in various plants (e.g., Arabidopsis, rice, tomato, wheat, cucumber, citrus, cotton).^10-12^^,^^29^^,^^30^ However, some studies have shown that under certain environmental stresses (e.g., salt stress, drought), the expression of these structural genes is not always stable. For example, Czechowski et al.^5^found that *ACT* expression fluctuated under drought stress in Arabidopsis, suggesting that *ACT* is not always an ideal reference gene under such conditions. Therefore, when *ACT* and TUA are selected as reference genes under specific stress or conditions tested, further validation of their expression stability is required.

**Table 1.** Overview of reference genes and their relative stability under different stresses.

Reference gene	Main function	Representative species	Stability under stress conditions (Summary)	Recommended use context	Key observations/controversies	References
*ACTIN*	Cytoskeleton, cell division	Arabidopsis, tomato, rice	Often unstable under biotic stress (e.g., pathogen infection); variable under drought; moderate stability under abiotic stress	Not recommended for pathogen or wound studies	Frequently overused despite known instability; some studies report acceptable performance in non-stressed tissues	[5,31,32]
*GAPDH*	Glycolysis, energy metabolism	Arabidopsis, tomato	Down-regulated under salt, drought, osmotic stress; highly variable in stress conditions	Avoid in abiotic stress experiments	Despite common use, multiple studies show poor stability; may be stable in control conditions only	[5,12]
*EF1α*	Protein synthesis (translation elongation)	Grapevine, cucumber	Generally stable under drought, salinity; slight variation under heat stress	Suitable for drought/salinity studies	High consistency across studies; one of the most reliable among classical candidates	[10,33]
*UBQ10*	Protein ubiquitination, protein turnover	Tomato, Rice	Broadly stable across diverse stresses; minor fluctuations under prolonged heat	Preferred for multi-stress or long-term experiments	Consistently ranked among top stable genes in qPCR validation studies	[34,35]
*18S rRNA*	Ribosome assembly translation	Peach, zucchini	Stable in many conditions, but shows variability under nutrient deficiency and extreme temperatures	Use with caution in nutrient-limited or low-light environments	Often overestimated as universal reference; not suitable for gene expression normalization in metabolic studies	[36,37]

*GAPDH* and *EF1α* are also commonly used reference genes, particularly in rapidly metabolizing plant cells.^10^^,^^15^ *GAPDH* is involved in glycolysis and plays a crucial role in cellular energy metabolism, so it is considered to have relatively stable expression under various tissues and treatment conditions. *EF1α* acts as an elongation factor in protein translation, aiding the transfer of amino acids, and is a necessary component of protein synthesis. Li et al.^15^ found that *GAPDH* and *EF1α* exhibited relatively stable expression levels under different stress conditions in celery. In addition, ribosomal RNA genes (e.g., *18S* and *26S rRNA*) are also commonly used as references owing to their high abundance in cells and are usually considered to express stably. However, recent studies have shown that ribosomal RNA expression fluctuates under stress conditions in some plants, indicating that ribosomal RNA genes also need to be validated when used as reference genes.^38^

## 4 Challenges in selecting plant reference genes

The stability of plant reference genes is crucial for accurate gene expression analysis. However, differences in different conditions, plant tissues, and species significantly affect the stability of reference genes. Therefore, selecting appropriate reference genes under different conditions remains a major challenge in plant molecular biology research. We analyzed the impact of stress conditions, tissue and developmental stage differences, and interspecies differences.

### 4.1 Impact of stress conditions

When plants are subjected to different types of stress (e.g., salt stress, drought, low temperature, and pathogen infection), complex physiological and biochemical responses are triggered, leading to significant changes in gene expression patterns.^5^^,^^12^ Therefore, the expression of common reference genes was unstable under these stress conditions. For example, *GAPDH*, a metabolic enzyme gene, shows relatively stable expression under normal growth conditions, but studies have shown that its expression is significantly affected under salt and drought stress.^6^^,^^16^ Additionally, traditional reference genes such as *ACT* and *18S rRNA* also exhibit expression fluctuations when plants face abiotic stresses.^29^^,^^33^ These examples highlight the need to optimize the choice of reference genes under specific stress conditions.

Under drought, salinity, and pathogen stress, the stability of traditional reference genes such as *ACT*, *GAPDH*, and *18S rRNA* often decreases.^6^^,^^16^ In contrast, *EF1α* and *eIF4α* has shown superior stability across multiple stresses in celery and cucumber.^10^^,^^15^ Therefore, condition-specific validation has become the most reliable strategy.

### 4.2 Differences in tissues and developmental stages

There are significant differences in gene expression patterns across different tissues and developmental stages in plants. The expression levels of the same gene vary between tissues or developmental stages, which limits the applicability of common reference genes. For example, in a study on rice development, genes such as *ACT* and *GAPDH* exhibited differences in expression at different developmental stages, making them less suitable as reference genes in rice tissues at different stages of development.^6^ Similarly, in Arabidopsis, some studies have shown that 18S rRNA expression varies between leaves and roots, potentially affecting its stability as a reference gene in different tissues.^5^

To address the impact of tissue and developmental stage on reference gene selection, researchers typically need to screen and validate reference genes suitable for specific tissues or developmental stages before the experiment. For instance, Tong et al.^37^found that *TEF2* maintained stable expression during fruit development in peach, making it an ideal reference gene. Additionally, Saha et al.^39^ systematically screened and identified a set of specific reference gene combinations in millet leaves, stems, and roots to ensure accuracy in tissue-specific expression analysis. In conclusion, owing to variations in gene expression across tissues and developmental stages, appropriate reference gene selection should consider the specific tissues or developmental stages involved in the experiment to improve the reliability of expression analysis.

### 4.3 Interspecies differences

The diversity of plant species means that a reference gene that is stable in one species exhibits unstable expression in another.^29^^,^^30^^,^^33^ Differences in gene expression regulation, metabolic pathways, and physiological states across species limit the stability of reference genes across species. For instance, *ACT* and *TUB* are widely used in Arabidopsis and rice, but in other species (e.g., tomato), their expression stability differs.^11^^,^^12^ Additionally, with the increasing number of non-model plant genomes being sequenced, researchers have found that some reference genes that are stable in model plants show significant fluctuations in new species.^35^^,^^36^ For example, in potato research, traditional reference genes such as *ACT* and *GAPDH* show expression differences under certain environmental conditions, suggesting that they were not ideal reference genes in all species.^40^

## 5 Recent advances in reference gene selection

With the development of genomic sequencing technologies and molecular biology tools such as quantitative PCR, progress has been made in the selection of reference genes. The expression stability of reference genes under different conditions and plant types is crucial for ensuring the accuracy of gene expression analysis. The stability of reference genes varies depending on the plant type and specific conditions, making it necessary to select appropriate reference genes tailored to specific plants and conditions.

### 5.1 Reference gene selection in model plants

Model plants, such as Arabidopsis and rice, are widely used in plant molecular biology research, and the selection of reference genes in these plants has been studied in depth.^41^ Arabidopsis is an important model plant in molecular plant research, and studies have shown variations in the expression stability of its traditional reference genes under different conditions.^5^ Czechowski et al.^5^ performed a high-throughput transcriptomic analysis and identified a set of reference genes suitable for various conditions, such as *UBQ10* and *EF1α*. This data-driven large-scale screening method has improved the reliability of reference gene selection. Additionally, in rice, Jain et al.^6^ conducted a comparative analysis of the stability of multiple reference genes and found that *EF1α* was relatively stable under salt and drought stress, making it suitable for use as a reference gene in qRT‒PCR. In recent years, RNA-seq has provided quantitative expression data for thousands of genes simultaneously. By computing expression variance (CV values) across datasets, researchers have identified stably expressed genes.^5^^,^^14^ These approaches outperform traditional candidate-gene screening, particularly under complex stress combinations.

### 5.2 Reference gene selection in crops

Owing to the diversity of growing environments and cultivation conditions, the selection of reference genes becomes an important challenge for gene expression analysis in staple crops. However, significant progress has been made in the selection of reference genes for crops such as wheat, maize, and soybean (Table 2). For example, in rice, Moraes et al.^42^ analyzed the stability of reference genes in different tissues and stress conditions and found that *EF1α* and *UBQ5* exhibited good stability under various environmental conditions, making them suitable for gene expression analysis. Similarly, in studies on drought stress in wheat, researchers have reported that *CJ705892*, *ACT*, and *UBI* exhibit high expression stability, making them suitable reference genes.^43^

**Table 2.** List of stable reference genes identified in major crops.

Plant species	Stable reference genes	Experimental conditions tested	Key functional roles & stability drivers	Performance summary & recommendations	References
Rice	*EF1α, UBQ5, eIF4A*	Germination, salt stress, heat stress, developmental stages	Involved in translation initiation and protein synthesis; generally conserved expression across tissues and stresses	Highly recommended for abiotic stress studies; EF1α and UBQ5 show consistent stability across multiple conditions	[42,44]
Maize	*EF1α, EIF4A*	Abiotic stress, hormone treatments, pathogen infection	Core components of translational machinery; low regulatory plasticity under stress	Reliable across diverse treatments; suitable for multi-stress or hormonal studies	[45,46]
Wheat	*Ta2291, TaFNRII, CJ705892*	Drought and nutrient treatments	Unknown functions (some are novel); may be regulated by stress-responsive pathways	Novel genes with broad stability; promising candidates for future validation in related cereals	[7,43,47]
Soybean	*TIP41, F-box*	Salt, drought, heat, ABA treatments	TIP41: involved in vesicle trafficking; F-box: ubiquitin ligase component	Both exhibit minimal expression variation under stress; potential for use in legume stress research	[23,48]
Cotton	*UBQ14, PP2A1, UBQ7*	Tissues and developmental stages	Ubiquitination-related (UBQ), phosphatase activity (PP2A)-essential housekeeping functions	Strongly stable across developmental and environmental gradients; ideal for gene	[49,50]

In soybean research, owing to the complex biotic and abiotic stresses it faces, the selection of reference genes has been more extensively studied. Hu et al.^48^ compared the expression stability of multiple reference genes under various stress conditions and found that *TIP41*, *UKN1* and *UKN2* exhibited stable expression when the light quality was changed. Additionally, new analytical tools, such as Ref-Finder and GeNorm, have been applied to the selection and stability analysis of crop reference genes. These tools use multifactor analysis of gene expression data to help researchers more accurately select appropriate reference genes, thereby improving the reliability of experiments.^2^^,^^51^

### 5.3 Reference gene selection in horticultural crops

Horticultural crops such as tomatoes, grapes, and strawberries exhibit gene expression differences across different tissues and developmental stages. Therefore, selecting suitable reference genes is particularly important in the study of horticultural crop species. For example, during fruit development in tomatoes, gene expression differences across different developmental stages are significant. ExpósitoRodríguez et al.^11^ compared the expression of multiple reference genes at different fruit development stages and found that *TIP41* and *SAND* exhibit high expression stability during fruit development, making them ideal reference genes.

In grapevine, owing to the differences in stress responses among different cultivars, reference gene selection has become a major issue. Studies have shown that *GAPDH* and *EF1α* exhibit good expression stability across different tissues and stress conditions in grapes, making them widely used for gene expression normalization in grapes.^13^ Furthermore, in the tissue-specific expression analysis of strawberries, *GAPDH* and *ACTIN1* exhibit consistent expression levels, providing reliable references for strawberry gene function studies.^52^ These studies indicate that reference gene selection for different horticultural crops needs to consider the specific tissues and developmental stages involved to ensure the accuracy of gene expression analysis.

## 6 Selection of new reference gene in plant species

**Figure 1.**
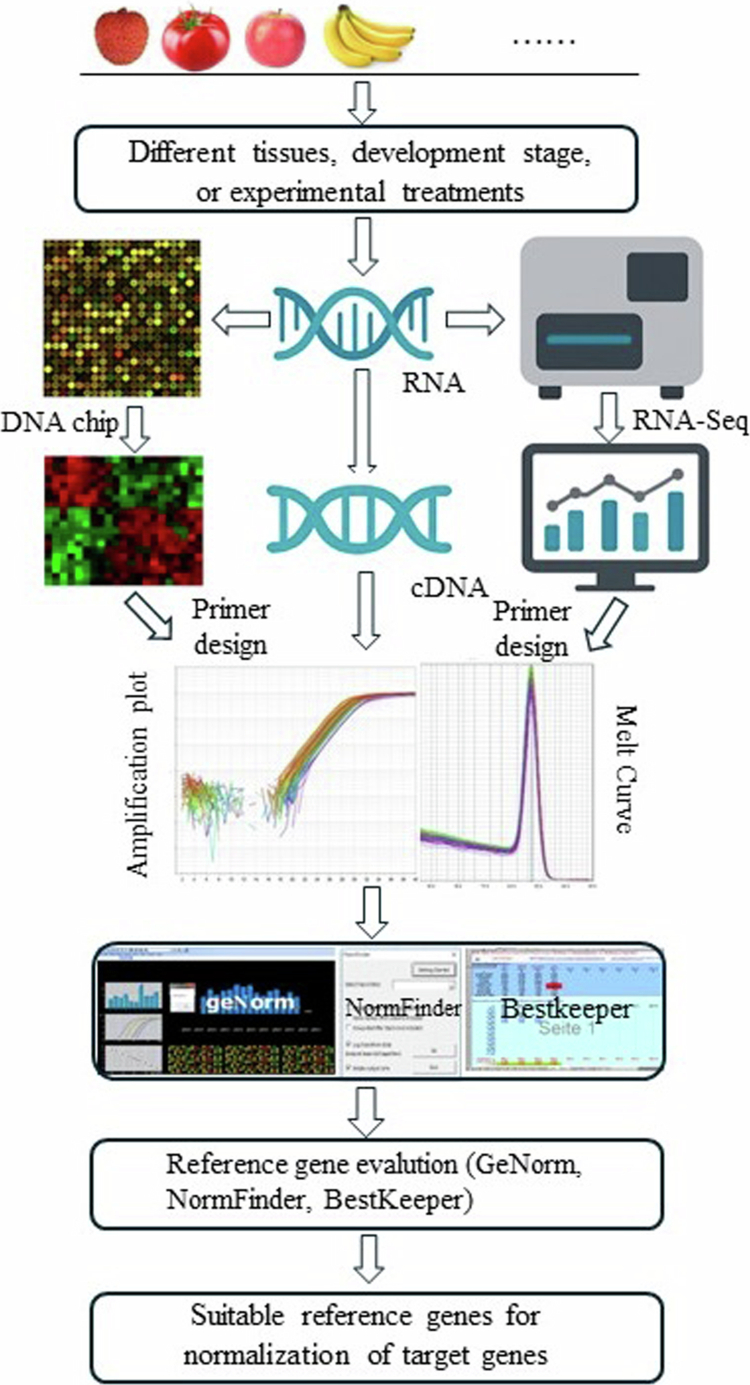
Identification of suitable reference genes for qRT-PCR normalization. Biological samples from different tissues, developmental stages, or experimental treatments are collected. RNA is extracted and analyzed by either a DNA microarray or RNA-Seq. Candidate reference genes were selected, and specific primers were designed for qRT-PCR. Amplification plots and melt-curve analyses were performed to verify primer specificity and amplification efficiency. The stability of the candidate reference genes was evaluated using GeNorm, NormFinder, and BestKeeper. Based on these analyses, the most stable reference genes are identified for accurate normalization of target gene expression.

With the development of high-throughput sequencing and multi-omics technologies, reference gene selection methods have continuously innovated, offering greater accuracy for plant gene expression analysis. Gene chip/EST-based screening and the application of multi-omics methods provide new perspectives on reference gene selection, making the process more scientific and efficient (Figure 1). Traditional reference gene selection typically relies on the validation of a few candidate genes, but these genes often exhibit significant variability in expression stability under different conditions and in different plant species. In recent years, RNA-seq has revolutionized reference gene screening. By analyzing transcriptomic data, researchers have identified reference genes that exhibit stable expression across various conditions and tissues. For example, through transcriptomic data analysis, Czechowski et al.^5^ identified a set of reference genes in *Arabidopsis* that exhibited stable expression under different stress conditions. This large-scale screening approach evaluates the stability of candidate reference genes by calculating the coefficient of variation across different samples, thus avoiding the instability caused by single reference genes influenced by specific conditions.

In important crops such as pepper, transcriptomic technologies have also been widely applied to reference gene screening. For example, Cheng et al.^53^ screened a set of stable reference genes, such as *CaREV05* and *CaREV08*, in chili peppers under different growth stages and stress conditions using RNA-seq data. This data-driven screening method effectively covers different growth environments and different treatments, improving the generalizability and reliability of reference genes. Moreover, transcriptomic analysis also reveals differences in expression stability between tissues and developmental stages, making the selection of tissue-specific or stage-specific reference genes more precise.

## 7 Future perspectives

In recent years, the methodological landscape for reference gene (RG) selection has shifted from small-scale empirical testing toward multi-omics-driven discovery. Classical tools such as geNorm, NormFinder, and BestKeeper evaluate expression stability within a limited gene set, but they are constrained by small sample size and experimental context. In contrast, transcriptome-wide and multi-omics analyses can now systematically identify stable candidate RGs by integrating gene expression variability across tissues, developmental stages, and stress conditions. For instance, transcriptome meta-analysis frameworks and databases such as Genevestigator’s RefGenes enable users to retrieve condition-specific RGs across thousands of RNA-seq or microarray datasets.^19^ The recently developed RGeasy web platform further operationalizes this approach by integrating transcriptomic data with statistical algorithms to automatically select RGs across multiple conditions.^54^ Moreover, integrative multi-omics studies that combine transcriptome and proteome layers have demonstrated that consistent expression profiles across data types improve RG reliability. These advances illustrate how data integration enhances reproducibility and reduces subjective bias in RG selection.^55^

Looking forward, artificial intelligence (AI) and machine learning (ML) are expected to transform RG identification into a predictive modeling process. A feasible computational pipeline could involve: (i) constructing a multi-omics compendium encompassing transcriptome, proteome, and epigenome data for the target species; (ii) applying ML algorithms such as random forest, support vector machine, or Lasso regression to evaluate expression stability based on quantitative features (e.g., coefficient of variation, expression breadth, network centrality, and tissue-specificity scores); and (iii) predicting context-specific RG panels optimized for distinct conditions (e.g., “salt-stressed leaves” or “flowering tissues”). Studies in human and plant transcriptomics have demonstrated that ML models outperform traditional stability metrics by integrating multidimensional data.^41^ Deep-learning-based multi-omics frameworks are also emerging to identify invariant expression signatures across stress treatments.^56^ In plant systems, combining these models with RNA-seq meta-analysis tools such as RefGenes and RGeasy could yield automated, interpretable, and condition-aware RG recommendations. We predict that future pipelines will integrate network-aware learning to penalize co-regulated genes, and transfer-learning architectures to extrapolate RG stability from model plants to less-studied species – ushering in a new era of data-driven, scalable, and reproducible reference gene selection.
